# Probing the functional and therapeutic properties of postbiotics in relation to their industrial application

**DOI:** 10.1002/fsn3.3465

**Published:** 2023-06-06

**Authors:** Abrar Asif, Muhammad Afzaal, Hina Shahid, Farhan Saeed, Aftab Ahmed, Yasir Abbas Shah, Afaf Ejaz, Samia Ghani, Huda Ateeq, Mahbubur Rahman Khan

**Affiliations:** ^1^ Food Safety and Biotechnology Lab, Department of Food Science Government College University Faisalabad Faisalabad Pakistan; ^2^ Women Medical Officer District Head Quarters (DHQ) Hospital Vehari Vehari Pakistan; ^3^ Department of Nutritional Sciences Government College University Faisalabad Pakistan; ^4^ Faculty of Pharmaceutical Sciences Government College University Faisalabad Punjab Pakistan; ^5^ Department of Food Processing and Preservation Hajee Mohammad Danesh Science & Technology University Dinajpur Bangladesh

**Keywords:** metabolites, microbiota, postbiotics, prebiotics, probiotics, symbiosis

## Abstract

Functional foods are gaining significant research attention of researchers due to their health‐endorsing properties due to their bioactive components either living cells (probiotics) or nonviable cells (prebiotics). The term “postbiotic” specifies the soluble substances, such as enzymes, peptides, teichoic acids, muropeptides derived from peptidoglycans, polysaccharides, cell surface proteins, and organic acids, that are secreted by living bacteria or released after bacterial lysis. Due to various signaling molecules which may have antioxidant, immunomodulatory, antiinflammatory, antihypertensive, and antiproliferative activities, postbiotics offer great potential to be used in pharmaceutical, food, and nutraceutical industries, to promote health and ailment prevention. This recent review is a landmark of information relevant to the production of postbiotics along with salient features to use in various fields ranging from food to immunomodulation and selective and effective therapy. It also puts forward the concept that postbiotics are way more effective than probiotics in the veterinary, food as well as medical field which ultimately helps in reducing the disease burden along with human health.

## Graphical abstract

Postbiotics are way more effective than probiotics in the veterinary, food as well as medical field which are ultimately helps in reducing the disease burden along with human health

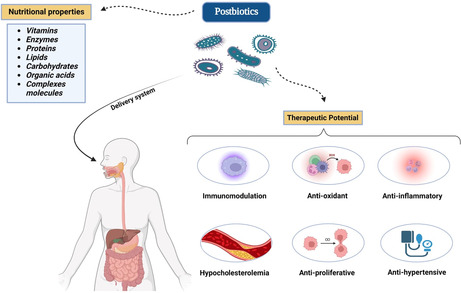

## 1 INTRODUCTION

The term gut microbiota is usually referred to live microorganisms present in human gut, its genomic sequence, and metabolites, along with its living environment are crucially important for gut microbiome balance (Przybyciński et al., 2023). The microbial community can enter the human body through the parts which are in direct contact with the external environment (e.g., the urogenital tract, upper respiratory tract, or skin). However, the gut microbiome is abundant in the gastrointestinal tract (Sorboni et al., 2022). A study developed a synbiotic relationship with the intestinal microflora in infants of age 3 years (Rodríguez et al., 2015). Human body needs essential nutrients to stabilize gut microbiota, as a return benefit, and consequently, healthy gut impacts several advantages over human body functions including immunomodulation, enhanced viability of nutrients, reduction in the oxidative stress and growing ability of microflora, and nourishing the integrity of the intestinal barrier (De Souza Valente & Wan, 2021; Monteagudo‐Mera & Charalampopoulos, 2018). These advantageous interactions between the GIT and microbial flora can be locally observed as well as in distant organs, due to the systemic placement of substances and cells that are fabricated in the intestine. This phenomenon is termed the gut–organ axis, based on which there is a distinction between the gut–skin, gut–brain, gut–lung axis, and many others.

Multiple factors play an important role in the microbial composition beginning from the prenatal phase, involving maternal gut microbiota make‐up, the delivery mode and kind of food consumed by the mother, antimicrobial therapy, and stressful conditions (Kumbhare et al., 2019; Łubiech & Twarużek, 2020). Furthermore, numerous studies have revealed that dysbiosis (an abnormal variation in the microflora of the intestine) can lead to allergic or autoimmune disease development (e.g., IBS i.e., inflammatory bowel syndrome, diabetes mellitus, and multiple others), neoplasm, and psychiatric disorders (Edwards et al., 2017). Fundamentally, curative approaches and formulations that impact microbial composition, and hence, the patient's health, have become progressively in demand (Edwards et al., 2017). The use of probiotic bacteria is a part of human civilization since early times and the postbiotic conception relies on the consideration that the valuable out‐turns of the microbiota are due to the secretion of various metabolites. Gut microbiota from healthy infants who are on mother feed was dominated by rods with bifidobacteria (bifid shaped) which seems to be absent in infants on formula milk hence suffering from diarrhea. This review settled the concept that microbiota plays a dominant role in sustaining health but the explicated Health effects of microbial flora were distinguished clearly after Metchnik off research in 1907 which associated Bulgarian farmers' prolonged lives with their fermented dairy food intake (Kechagia et al., 2013). In 1953, the word “probiotics” was used to narrate “essential active substances for the healthy development of life” (Dhruv et al., 2021). Because of the existing prescription of probiotics for the prevention and treatment of numerous viral diseases, probiotic supplements have recently been recommended as just an adjuvant therapy for COVID‐19 (Conte & Toraldo, 2020; Olaimat et al., 2020; Patra et al., 2021; Santacroce et al., 2021).

In this regard, probiotics' basic processes include reducing bacteria adhesion, improving gut barrier performance, and enhancing immunological functions (Stavropoulou & Bezirtzoglou, 2020). By adhering to the virus, probiotic microbes can prevent viral activity and virus penetration in host tissue (Hamida et al., 2021). Research on Functional food has considerably advanced in the previous few decades. The terms “synbiotics” and “prebiotics” were popularized by Gibson and Roberfroid, who investigated them in 1995 to explain food supplements that are indigestible by the host but exert a favorable impact by selectively stimulating the activity or growth of intestinal microbiota. This terminology was further improved by the skillful team of the International Scientific Association for Probiotics and Prebiotics (ISAPP) to narrate prebiotics as “a substrate used by host microbiota selectively to grant beneficial effects to health” (Gibson et al., 2017; Mayorgas et al., 2021). In the meantime, the word symbiotics were collected to elucidate the probiotics and prebiotics relationship that assists the host by promoting microbial colonization and survival in the gut (Vrese & Schrezenmeir, 2008). Additionally, a study revealed that the use of prebiotics, probiotics, and synbiotics (a combination of prebiotics and probiotics) could influence the yields of chemical messengers (hormone levels and neurotransmitters) and inflammatory components, interfering with the eating plan which leads to obesity (Mohd Fuad et al., 2022).

Recent studies give rise to new terminologies based upon nonviable microorganisms or bacterial metabolic byproducts which likewise impose bioactivity on the hosts, and these terms include postbiotics and paraprobiotics (Taverniti & Guglielmetti, 2011) which are introduced in the literature to describe such bioactive components which do not fit the old descriptions (prebiotics, symbiotics, and probiotics), to elucidate a probiotic's curative effect in a specified system or ailment, as in psychobiotic situation (Dinan et al., 2013). Postbiotics are substances that are metabolic byproducts or products processed by live microbe or liberated after bacterial nonviability, and they are further termed as biogenic, metabolites/CFS (cell‐free supernatants), or metabiotic. By enhancing bioactivity, these metabolic byproducts give an advantage to the host (Cicenia et al., 2014). Bioactive constituents, for example, probiotics, prebiotics and postbiotics, on the other hand, have become progressively important to researchers in the past few decades through modifying the composition of gut microbiota, immunological reactions, colorectal cancer (CRC) cure efficiency and lowering CRC biomarkers (Ejtahed et al., 2012). *Bifidobacterium* spp., certain gram‐negative strains (*Escherichia coli Nissle 1917*) (Behnsen et al., 2013), lactic acid bacteria (LAB), and yeasts are examples of probiotic microflora. The LAB are wide collection of microbes that are both gram‐positive and catalase‐negative. The most valuable and common genera are *Streptococcus*, *Lactococcus*, *Lactobacillus*, and *Leuconostoc* found in traditional LAB (Zheng et al., 2020). For preparing functional foods, postbiotics from *Bifidobacterium breve* and *streptococcus* are used due to their evaluated efficiency in clinical trials. These postbiotics decreased the signs of breathing allergy and suggested food in the initial age of children with progressive history of atopy (Żółkiewicz et al., 2020). Often used probiotic microorganisms are *Bifidobacterium* spp. and *Lactobacillus* spp. Those generally regarded as safe (GRAS) agents are the colon's predominant and helpful bacteria, and they may be amplified in the gut without causing any serious negative effects. As possible probiotic bacteria, LAB and yeasts are also employed (Fijan, 2014) in functional items such as those made using kefir cultures (Erdogan et al., 2019).

Currently, to overcome some challenges regarding probiotic cell utilization, many effective alternatives such as prebiotics (dietary fibers) and postbiotics (microbial‐derived biomolecules) have been used to stabilize the gut microbiota composition and, as a result, to develop eubiotic situations, which resulted in the formation of homeostasis (Homayouni Rad et al., 2020). In this regard, there is strong evidence that the gut microbiota's significant health‐promoting impacts are related to their nonviable byproducts called postbiotics. However “postbiotics” is novel, and it has quickly gained acceptance in the field of food science, as well as in human health and nutrition, drawing attention to their possible future use as nutraceuticals, functional foods, and pharmaceuticals in the biotechnological and food industries along with pharmaceutical manufacturing industries (Homayouni Rad et al., 2020).

In the preparation of foods by fermentation, different Lactobacillus strains are utilized as prime and subsidiary starters (probiotic and protective strains). Some well‐known probiotic products employ probiotic strains such as *L. casei and L. acidophilus*. (e.g., BIO, Actimel, LC1, and Yakult). Soluble consumable oligosaccharide carbohydrates (Fructo‐oligosaccharides and galacto‐oligosaccharides) and some other substances, commonly referred to as prebiotics, may be given to the diet to encourage probiotics to thrive in the intestine and generate bioactive molecules (Markowiak & Śliżewska, 2017). The unsuitability of live starter/probiotic cultures with various matrices and conditions, which inhibits their survival and growth in food is the main problem of using them directly on food. The use of postbiotics, as a substitute, prevents trouble interacting among primary and secondary starters, as well as the diet (Pujato et al., 2014). Generally, it is considered that postbiotics own numerous appealing properties involving clear chemical structures, prolonged shelf life (up to 5 years, when utilized as an ingredient in beverages and food or as nutritional supplements), and safety dose parameters that are greatly called on (Tomar et al., 2015). Additionally, it has been illustrated that postbiotics can imitate the healthy benefits of probiotics which results in the circumvention of essential administration of viable microbiota, which may not always be safe as formerly manifested by Tsilingiri et al. (2012). As a result, instead of utilizing a living bacterium, postbiotics may take full use of its wide‐ranging antibacterial action, combinatorial activity among organic acids as well as other metabolites, and the postbiotics mixture's excellent thermal conductivity (Moradi et al., 2020). Postbiotics are preferred over probiotics in large factories due to their specified configuration, stability, convenience of utilization and preservation, durability across a wide temperature and pH range, and wide‐ranging antibacterial action (Barros et al., 2020). Additionally, Shenderov (2013) performed an investigation that showed that postbiotics have suitable pharmacokinetic properties (ADME), which could stipulate a higher extent to signal host tissues and multiple organs thus evoking numerous biologic responses.

In inflammatory GI diseases, postbiotics have been offered as food supplements to enhance intestinal homeostasis instead of probiotics where the existence of microbe‐associated molecular patterns (MAMPs) that may trigger natural defenses as well as encourage inflammation makes the consumption of probiotics a risk for humans (Mayorgas et al., 2021). There are many postbiotics present with different uses other than food. But, their way to prepare and analyze is little identified, as well as the factors that influence the production of every postbiotic substance, which could be a hurdle to new research and large‐scale application in the food industry. The use of mixture of postbiotics and individual postbiotics in food safety, biotherapy and functional foods (Nataraj et al., 2020) nutrition (Wegh et al., 2019), disease control (Malagón‐Rojas et al., 2020) animal feed and drug companies (Cuevas‐González et al., 2020) and allergy treatment (Homayouni Rad et al., 2021) has recently been revised.

## 2 RELATION OF PARAPROBIOTICS, POSTBIOTICS, AND PSYCHOBIOTICS

Keeping in view the actual definition of probiotics, the survivability of bacterial cells is a requirement for the attainment of a positive impact on the health of the host. Nevertheless, after the realization of some processes and clinical effects that were not directly linked with live microbiota, the previous model got shattered. Resultantly, new terms like paraprobiotics and postbiotics were introduced. These terms describe how nonliving bacterial cellular structures, microbial remnants, or cellular debris, when given in the right doses, can act as health or well‐being promoters with extra biological activity (Aguilar‐Toalá et al., 2018). Paraprobiotics, also termed as “inactivated probiotics, non‐viable” or “ghost probiotics,” are described as “non‐viable microbial cells (either intact or ruptured) or raw cellular extracts (with complex chemical composition) that granted certain benefit to the consumer when taken in sufficient quantities” (Barros et al., 2020). Consequently, these microorganisms have compromised survivability due to mechanisms that have encouraged structural and metabolic changes in bacteria (de Almada et al., 2016). Due to its meaning, Tavertiti & Guglielmetti (Taverniti & Guglielmetti, 2011) coined the term “paraprobiotics.” The word “para” means “side by side” or “atypical” in ancient Greek (as frequently employed in the literature of Chemistry along with the words meta and ortho), which may suggest both commonality and difference from standard probiotic definitions.

Terms like “metabiotics,” “postbiotics,” “biogenic,” “probiotics cell fragments”(PCFs), and metabolites/CFS—Cell's free supernatant were coined to describe bioactive soluble factors with a molecular weight of 5–100 kDa, generated by living probiotic bacteria or removed after cell rupture that gives any physiological health benefit on the host (Aguilar‐Toalá et al., 2018). These are the “compounds generated by microorganisms, liberated from microbial materials or food and include nonliving cells, that encourage health and welfare when directed in enough quantity. Excessive preclinical studies showed that taking supplements with probiotics can improve development, process, and intellectual actions via the gut–brain axis (Barros et al., 2020). The term “psychobiotic” was proposed to describe the specie of probiotic bacteria with psychrotrophic properties, which act as transporters of neuroactive substances. Psychobiotics were described by Dinan and coworkers in (Dinan et al., 2013) as live microorganisms that, if consumed in sufficient amounts, give mental fitness advantages through connections with the intestinal microbiota. Reports reveal that “psychobiotic” modulate the neurotransmitters and proteins, cognitive functions, mood, learning process, and memory (Cheng et al., 2019). Psychobiotics follow the mechanism of neurotransmitter production including catecholamines, GABA (gamma‐aminobutyric acid, acetylcholine, and serotonin), and regulate hypothalamic–pituitary–adrenal axis (HPA) in traumatic or anxious situations and antiinflammatory effects (Misra & Mohanty, 2019). Additionally, advancement in the research revealed that microbial flora of the gut plays a vital part in the conduct of brain and cognitive development. Hence, brain‐related disorders and ailments can be improved by causing variations in gut–microbial flora. As mentioned above, even though the “psychobiotic” term is based upon psychiatric criteria, we cannot characterize it as independent terminology and a novel class of probiotics because the exact mechanism of action is exactly relevant to the others like paraprobiotics, probiotics, and postbiotics. That is why the employment of this term imparts no logic and is just like the derivation of the terms “gutbiotic” or “gastroprobiotic” which certainly impact the GIT but without adding any novel impact to the existing terminologies (Zendeboodi et al., 2020).

Additionally, the term “parapsychobiotic” was generated to describe paraprobiotics that may help grant mental health (Nishida et al., 2017). Due to its anxiolytic and antidepressant effects, both terms, parapsychobiotic, and psychobiotic, were categorized into subclasses of paraprobiotics and probiotics. In contrast, it is critical to confine the usage of such logical terms to the academic group (Martín & Langella, 2019) for preventing unneeded public misunderstanding, as even the concept of probiotics is still a mystery to them.

## 3 PARAPROBIOTICS

The immunomodulatory mode of paraprobiotics is affected by the ways that are used to obtain them. These methods could be physical such as temperature, high hydrostatic pressure, ultraviolet and γ radiation, ultra‐sonication, and chemical deactivation such as acid deactivation or lyophilization (Aguilar‐Toalá et al., 2018). Even though there are numerous approaches, the most suitable strategy will be determined by the microorganisms utilized and the predictable clinical advantage (de Almada et al., 2016). As a result, a method must be chosen for inactivating the microbes while simultaneously preserving the probiotic's positive benefits. In humans and animals, many clinical and preclinical tests have shown the health advantages of paraprobiotics, including effectiveness in the inhibition of alcohol that can cause liver illness, breathing and toxic illnesses like atopic dermatitis, diarrhea, dental caries, colitis, pathogen inhibition, allergies, immune system modulation, and gut microbiota modulation (Aguilar‐Toalá et al., 2018). Trindade et al. (2021) studied paraprobiotics especially *Lacticaseibacillus rhamnosus CGMCC1.3724* effect of mucositis intestine by using murine model. They concluded that paraprobiotics change the mucositis physiology. It reduces the weight, reduce inflammation, increase intestinal secretion and change the Muc2 gene expression. Another study of Lim et al. (2022) prevents obesity and obesity‐induced inflammatory responses through paraprobiotic Lactiplantibacillus plantarum K8. The majority of these studies focus on the direct intake of paraprobiotics in suspension form. However, as shown in Table 1, researchers considering the utilization of food as a transport vehicle for paraprobiotics has gained interest in recent years.

**TABLE 1:** Examples of para probiotics that are used in meals to prevent probiotic bacteria from becoming inactive.

Probiotic	Food	Method to inactive	Beneficial properties	References
*Lactobacillus gasseri* (CP2305)	Fermented milk	Heat treatment (95°C for 30 s)	Regulating intestinal function in people who have a proclivity for constipation	Sugawara et al. (2016); Yamada et al. (2020)
*Lactobacillus gasseri* (CP2305)	Fermented milk	Heat treatment (95°C for 30 s)	Influence on gastrointestinal function and environmental regulation	Sugawara et al. (2016)
*Lactobacillus gasseri* (CP2305)	Fermented milk	Heat treatment (95°C for 30 s)	Stress‐related symptoms are relieved, resulting in improved health	Nishida et al. (2017)
*Lactobacillus gasseri* (CP2305)	Isotonic beverage	Heat treatment	Provision for athletes' fatigue retrieval, anxiety alleviation, and depressive humor in difficult conditions	Barros et al. (2020)
*Lactobacillus gasseri* (CP2305)	Fermented milk	Heat treatment (95°C for 30 s)	Modifications in sleepy manners and gastrointestinal habits of individuals throughout harsh circumstances, as well as inhibit the extreme stress of sensitive gene	Barros et al. (2020)

## 4 POSTBIOTICS

In literature, for distinguish postbiotics several terms just as nonbiotic, parapsychobiotics, paraprobiotic have been studied (Salminen et al., 2021). Commonly, different fermented foods are the natural source of postbiotics that can be obtained from manufacturer strains in situ including bacterial and fungal species (Collado et al., 2019). Thus, in production processes including heat, enzymatic (Li et al., 2012), ultrasonication (Amaretti et al., 2013) and solvent extraction (Kim et al., 2011) treatments, cell rupture is used. Different techniques (centrifugation, dialysis, lyophilization, and column purification) are used for removal and cleaning procedures that are required for cellular products. According to experimented physiological advantages, postbiotics can be classified into different types and their creation. All these bacterial cell components and microbial activity can be used to deduce this, that is, metabolites synthesis and production resulting from the enzymatic action of microbiota over the food material (Table 2) (Collado et al., 2019). Because postbiotics do not need severe production or preservation and have been shown to replicate probiotic operations and processes, postbiotics are perfect for poor countries (Bourebaba et al., 2022). As a result, many of the health benefits gained from eating fermented foods are linked to postbiotics, because they include both eaten live microbes and microbial structure and metabolite generated during fermentation.

**TABLE 2:** Postbiotics: Their composition and physiological advantages.

Composition	Examples of postbiotics	Physiological advantages	References
Compounds produced by microbes	Peptidoglycan Polysaccharides Lipoteichoic acids Cell surface proteins	Immunomodulation	Collado et al. (2019)
		Antiproliferative	
Metabolites	Lactic acid	Antiinflammatory Immunomodulation Antimicrobial Antioxidants	Aguilar‐Toalá et al. (2018)
	Peptides/Proteins		
	Bacteriocins		
	Enzymes		
	Polysaccharides Organic acids Lipids (short‐chain fatty acids)	Antiproliferative Hypocholesterolemia	
Metabolites	Amino acids with branches	In the intestinal epithelium, BCAAs can control how defensins are expressed	Peluzio et al. (2021)
Enzymatic metabolites of microbial action	Due to the reaction with the water breakdown of milk casein releases peptides	Antihypertensive	Al‐Ishaq et al. (2021)
Enzymatic metabolites of microbial action	Cell membrane parts	Various immune cell immunologic reactions	Jung et al. (2022)

### 4.1 Classification of postbiotics

**FIGURE 1:**
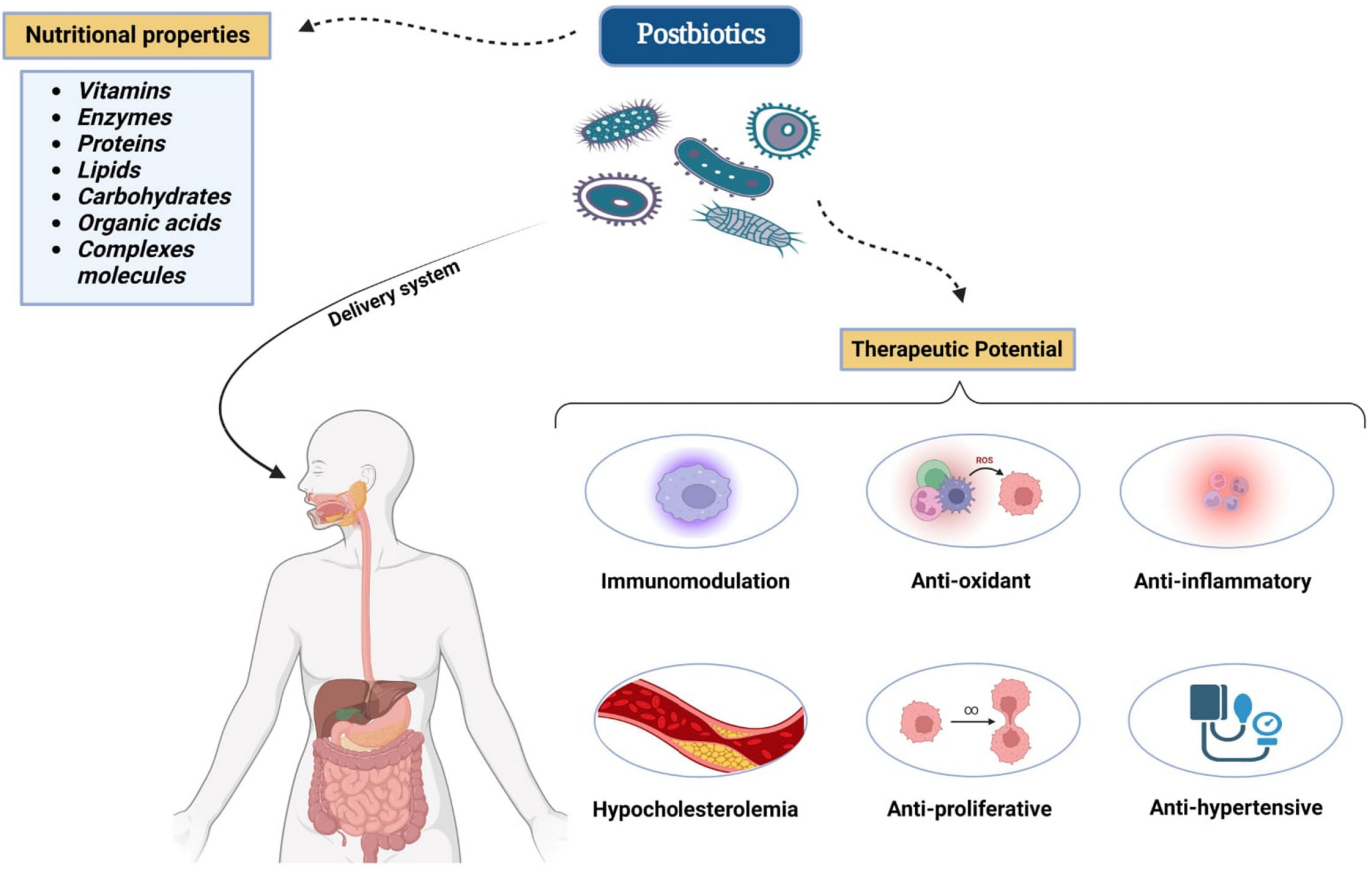
Postbiotics with health‐promoting properties are categorized.

According to composition of postbiotic, they are classified as proteins, lipids (SCFA), vitamins (Biotin, Riboflavin, ascorbic acid, etc.), organic acids and complex compounds just as lipoteichoic acids (Hernández‐Granados & Franco‐Robles, 2020; Park et al., 2022). They are also categorized by their bioactivities such as antihypertensive, antioxidant, immunomodulation, hypocholesterolemia, antiinflammatory, and antiproliferative effects (Figure 1). Along with the natural process of creating postbiotics, different laboratory techniques like high pressure, ultraviolet rays, thermal treatments, ionizing radiation, and sonication produced these postbiotics in pure form (Rad et al., 2021). Other methods for inactivating and producing postbiotics such as ohmic heating and supercritical CO2, pulsed electric field, pH changes, and drying, may also be effective (de Almada et al., 2016). The most often ways used for postbiotic preparation include heat treatments and formalin methods. These inactivation procedures will help preserve the beneficial microbial's health‐promoting characteristics (Dunand et al., 2019).

### 4.2 Preparation of postbiotics from LAB

The researchers made cell‐free supernatant solutions to obtain probiotics mixture that includes:

1. Microbial metabolic foods
2. Metabolites produced as a result of LAB's action on culture components
3. LAB‐produced structural components are utilized as a postbiotic combination (Nataraj et al., 2020).

The production of postbiotics (cell‐free supernatant) from LAB in different five steps by using extraction method.

**FIGURE 2:**
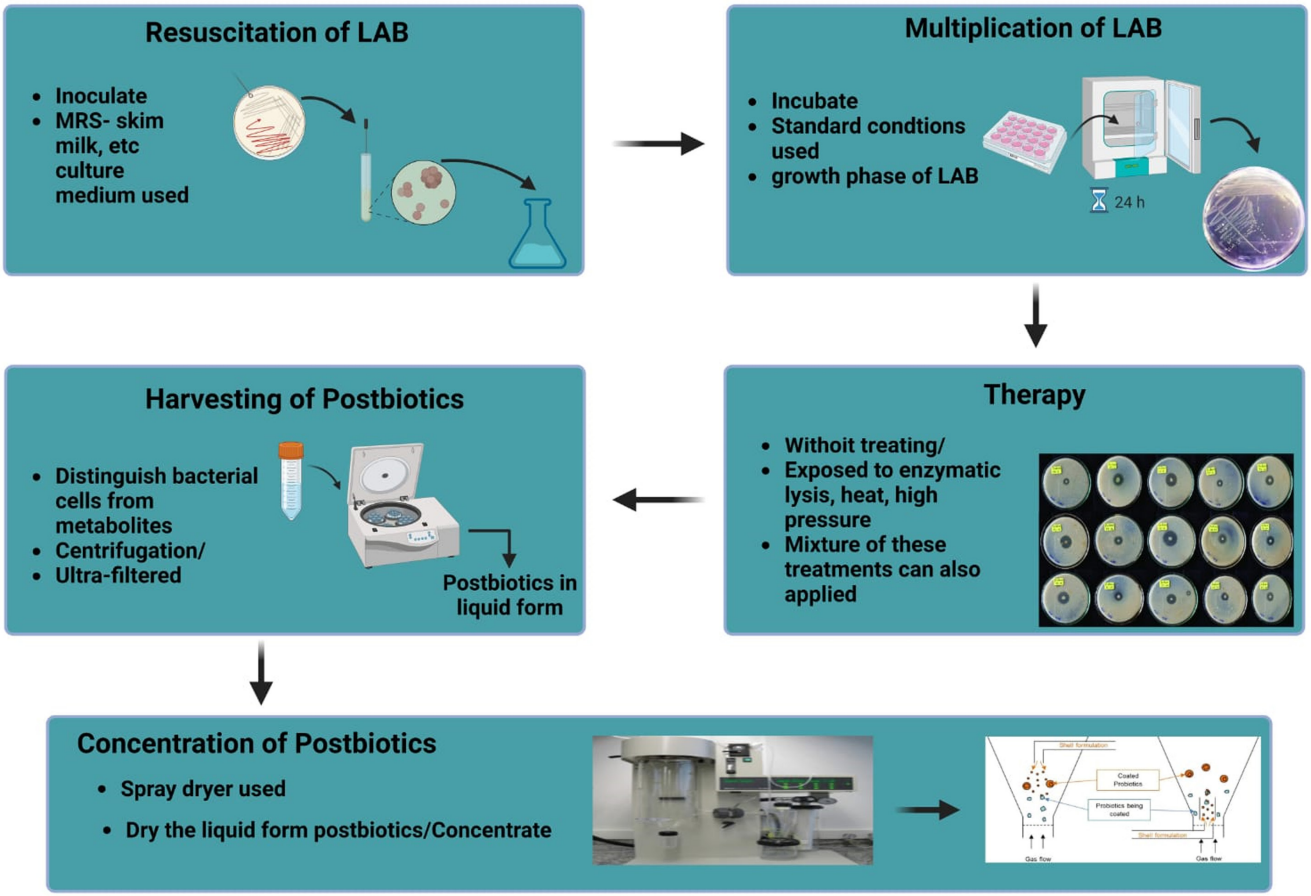
Lactic acid bacteria preparation by using MRS medium.

1: the resuscitation of LAB, 2: multiplication of LAB, 3: analysis, 4: harvesting of postbiotics, and 5: postbiotic concentration. When prepared in an MRS medium, it generates an acid fermentate which is free from bacteria with a brown to brownish yellow color (Moradi et al., 2019). The amount and kind of products achieved are primarily determined by the strain of bacterium, culture media along with bacterial treatment after propagation among other factors. As depicted in Figure 2, postbiotics mainly consist of soluble components such as metabolic by‐products or products that were released in media during the growth of bacteria (Żółkiewicz et al., 2020).

After proliferation, microbial cells were exposed to enzymatic lysis, heat, and high‐pressure methods, or a mixture of these treatments (Figure 2) (Cuevas‐González et al., 2020). These treatments add certain extra intracellular metabolites and cell wall resultant substances to the postbiotics mixes. The resulting mixtures are centrifuged or ultra‐filtered to distinguish bacterial cells from postbiotic metabolites in postbiotic mixtures, both untreated and treated (Cuevas‐González et al., 2020). At industrial level, for preparing postbiotics many aspects are needed to consider just as fermentation media, bacterial proliferation and concentration procedures. In general, LAB is used for postbiotic production as a primary or secondary starter. The most typical method for producing postbiotics is to culture the LAB in culture media, followed by an extraction phase (centrifuged at 4000–12000 g for 10 min at 4°C or dialysis) (Figure 2) (Dunand et al., 2019).

### 4.3 Identifying and counting methods of postbiotics for quality assurance

Postbiotics require extensive analysis for qualitative and quantitative recognition and characterization since they are made up of complex molecules with varying degrees of polymerization and glycosidic linkages. As their composition is based on complex molecular structures, revolutionized analytical approaches are needed and study goals play a vital role in the selection of appropriate instrumental technique (s) along with characterization type (quantitative and/or qualitative) followed (Barros et al., 2020). The isolation process can be completed using HPLC or capillary electrophoresis that permits component quantification. To analyze the qualitative and quantitative composition of postbiotics, HPLC is the most applied method (Moradi et al., 2021). However, colorimetric techniques, proton nuclear magnetic resonance, and spectroscopic techniques (liquid chromatography–tandem mass spectrometry (LC–MS/MS)) may all be used to determine the composition of complicated forms like oligosaccharides (Aguilar‐Toalá et al., 2018). In this method, proteolytic enzymes help to digest proteins and are then separated by LC–MS. In order to produce different protein expression profiles of *Lb. casei* throughout glucose hunger LC–MS/MS is used (Mbye et al., 2020). Moreover, proton‐based nuclear magnetic resonance (1HNMR) spectroscopy can also be employed to determine and quantify cell‐free supernatants (CFS) metabolites (e.g., amino acids, organic acids, monosaccharides, ketones, and alcohols) produced by different *Lactobacillus* strains (Fuochi et al., 2019). Meanwhile, extracellular metabolomics (Barros et al., 2020) can be used to identify and quantify worldwide main metabolites released throughout bacterial development that are an innovative approach consisting of many high‐efficiency analytical techniques and methods capable of monitoring metabolic level change.

## 5 APPLICATIONS AND USES

### 5.1 Technical features

As previously argued, the progress of probiotic products must adhere to specific guidelines for bacterial strains to survive big industrial production and maintain viability until consumed. However, many aspects such as the constituents of the food matrix such as carbohydrates, pH, lipids and protein concentrations, water activity, the occurrence of naturally occurring antibiotics, and the handling and storage conditions might reduce the probiotic cell viability during factory processing (Collado et al., 2019). Post probiotics and Para probiotics are more durable and safer at manufacturing purposes; therefore, their utilization in food items may provide food manufacturers with various technological benefits over the same live microorganisms. The identification and isolation of safer and functional strains of postbiotics are a main challenge for using postbiotics in products to determine the specific population and the specific dose (Thorakkattu et al., 2022).

### 5.2 Beneficial features

Postbiotics protect the health of the host against major infections caused by pathogenic microbes utilizing a variety of processes including pathogen adhesion inhibition, attack of pathogens, biofilm development, and enhanced immunomodulatory effects in Gut microbiota (Decuypere & Dierick, 2003). Extended research work showed the advantageous impact of Postbiotics obtained from *Bifidobacterium longum* (probiotic belonging to the *Actinobacteria phylum*) in minimizing intraluminal pH (Barrón‐González et al., 2019). Postbiotics generated from *Lactobacillus acidophilus* (*NCC 2581, NCC 2538, and NCC 2592*) halt cysts formation (Pérez et al., 2001). Many in vitro studies have been performed primarily on Lactobacillus strains because the postbiotics secreted by Lactobacilli seem to be a rich source of bacteriocins which hinders the growing ability and activities of divergent pathogenic organisms (Cicenia et al., 2014). Postbiotics obtained from Lactobacillus aids the immune system of the host by reducing the level of transaminase, decreasing TNF‐α, IL‐6, IL‐1β, IL‐8, and increasing intestinal expression of IL‐10, TLR4, IL‐12, IL‐10, active caspase‐3, claudin, occluding, mucin and MAP1LC3A (Wang et al., 2019).

In vitro and in vivo studies, postbiotics produced from *Leuconostoc mesenteroides* (*Lnm‐1RM3*) suppressed *Listeria monocytogenes* invasion and infection and can be used to boost the host's immune system's ability to fight infections caused by *Listeria* species more specifically *L. monocytogenes* (Nakamura et al., 2012). A research revealed that the supernatants of various *Lactobacillus* strains can obstruct *Streptococcus mutants*, hence improves dental caries (Rossoni et al., 2018) along with LAB, Bifidobacteria postbiotics impart positive effects on dental health which revealed from various studies. Furthermore, several postbiotics generated from Lactic acid bacterium (LAB) can give significant protection against virus‐induced illnesses. *Lactobacillus* species produced a vast metabolite variety such as gamma‐aminobutyric acid, lactic acid, and acetic acid, and have antibacterial (Di Cerbo et al., 2016) and antiviral effects (Sunmola et al., 2019). Gastric Corona inhibition, rotavirus in vitro, and HIV and a prominent reduction in viral load in vivo (Hasan et al., 2021) could play an important work in gastrointestinal disorder treatment.

## 6 THE IMPACT OF POSTBIOTICS IN IMMUNOMODULATION

There's an interaction between helpful gut bacteria and the health of the host, according to a growing body of data. In the past few years, research studies have shown that probiotics' postbiotics are responsible for a major portion of immunomodulatory effects (de Almada et al., 2016). The postbiotic agents fabricated from lactic acid bacteria can quickly engage with epithelium and immunological units to trigger inborn or innate immunity, providing the host with an instant effect as expressed in Table 3 (Taverniti & Guglielmetti, 2011). In this context, an in vitro investigation found that both viable probiotics and those produced from *L. gasseri TMC0356* can have acceptable immunomodulation response but surprisingly, the postbiotics produced a greater rise in IL‐12 release than the viable postbiotics. The unique chemical makeup of the postbiotic's cellular wall generated from *Lactobacillus rhamnosus HN001* increases leucocyte phagocytic activity, which promotes immunity (Gill & Rutherfurd, 2001). It is worth mentioning that postbiotic agents generated from various LAB strains have a wide spectrum of immunomodulatory effects.

**TABLE 3:** Biological activities of postbiotics in gastrointestinal disorders.

Species	Species having immunomodulatory effects	Postbiotics	Advantages	References
*Lactobacillus acidophilus* (LB, GG) & plantarum b240	*Lactobacillus acidophilus* A2, *L. gasseri* A5	Indeterminate, for Plantarum species; Heat‐killed cells, for reuteri species; cell‐free supernatant	Irritable bowel syndrome (IBS) sufferers' Diarrhea treatment, Prevention of necrotizing enterocolitis & Protection against Salmonella infection and translocation	Wegh et al. (2019)
	*Lactobacillus reuteri* CRL1098			
The mixture of Bifidobacterium, Lactobacillus, Lactococcus, and *Streptococcus thermophilus*		Intracellular content	Antioxidant activity in vitro	Amaretti et al. (2013)
*Lactobacillus rhamnosus* GG		Surface layer proteins	Reduced production of inflammatory cytokines results in antiinflammatory effects	Qi et al. (2020)
*Lactobacillus rhamnosus* KL37		Exopolysaccharides	Controlling immunological responses triggered by T cells in various inflammatory disorders	Nowak et al. (2020)
*Enterococcus faecium* and *Lactococcus lactis*		Cell‐free supernatant	Antiinflammatory and cell protection qualities	Dowdell et al. (2020)
Bifidobacterium spp.	Bifidobacterium bifidum BGN4	Cell‐free supernatant	Producing bacteriocins against pathogenic bacteria and yeasts	Amiri et al. (2021)
	And			
	Bifidobacterium breve M 16‐V	Sometimes Live and heat‐killed cells		

### 6.1 Postbiotics' impact on the treatment of colitis

The intestine's normal function disrupts by colitis (a chronic inflammatory disease) (Zheng et al., 2016) which can be prevent or delayed by few postbiotic agents, developed from bifidobacterial and lactobacilli strains and they have positive health effects on the host (Wasilewska et al., 2019). Those postbiotics produced from *Bifidobacterium bifidum* (*BGN4*) and *Bifidobacterium breve* (*M 16‐V*) exhibit notable inflammation‐reducing properties on peripheral mononuclear blood cells (Abbasi et al., 2021). The most appealing postbiotic possibilities for the prevention & treatment of gastrointestinal illnesses like inflammation in the colon, metabolic disorders, and cancers are tryptophan compounds like indole‐3‐propionic acid and short‐chain fatty acids (Yang et al., 2022).

### 6.2 Postbiotics and antidiarrheal effects

Diarrhea is a common gastrointestinal disease caused due to microbial and viral loads; however, radiotherapy and other traditional cancer treatments can produce diarrhea induced due to radiation therapy in radio‐oncology patients (Linn et al., 2019). Probiotics and their generated postbiotics continue to play a vital role in lowering the effects of diarrhea, according to advanced clinical research (Lai et al., 2019). A strong connection is observable between the utilization of postbiotic supplements generated from *Lactobacillus plantarum* (*b240*) and the reduced incidence of diarrhea (Abbasi et al., 2021). Changes in mucosal structure and an expanded population of favorable gut microbes could be the cause of these phenomena (Loh et al., 2014). In a clinical investigation on people treated with oral rehydration solution for diarrhea, it is found that adding a postbiotic powder generated from *Lactobacillus acidophilus* (*LB*) to the ORS (oral rehydration solution) shortened the duration of the condition to a single day (Liévin‐Le Moal et al., 2007). As a result, postbiotics can be presented as potential treatments for diarrhea that are free from major adverse effects. *Salmonella* induced inflammatory bowel disease (IBD) was demonstrated to be protected from postbiotic *Lactobacillus paracasei* (*B21060*) (Mohd Fuad et al., 2022).

## 7 APPLICATIONS OF POSTBIOTICS IN FOOD AND PHARMACEUTICAL INDUSTRIES

An advanced group of healthy products that have probiotics emerged as a result of an increasing understanding of functional foods. One issue with probiotic usage is the availability of antibiotic‐resistance genes in certain varieties, which can be transmitted to hazardous bacteria through gene transfer (Imperial & Ibana, 2016). Some other big difficulty with probiotics is keeping bacteria alive during manufacturing and storage just as the survival of probiotic strains in a delivery method can be affected by many factors, including interactions with other microbial species present, end acid content of the item, moisture content, temp, nutrient concentration, growth regulators and inhibitors, inoculum level, fermentation duration, chemical oxygen demand, and synthesis procedure just as spray and freeze drying, etc. (Santivarangkna, 2015). Additionally, variations in advertised and actual probiotic concentrations in various products for medical and veterinary use have been reported in the past (Weese & Martin, 2011). As a result, the deficiency of probiotic resilience may compromise the stated health advantages of probiotic supplementation. But at the other side, postbiotics are thought to be far more resilient than the bacteria from which they are derived (Venema & Van den Abbeele, 2013) found that antimicrobial peptides formed by *Bacillus* sp. strain *CS93* such as bacilysin and chloromethane are water soluble, thus suggesting that they could be used in many food products. Similarly, the usage of definite phytase‐producing lactic acid bacteria as bread starters has been stated as a better option for manufacturing low‐phytate whole wheat bread (Palacios et al., 2008). These chemicals would not be a problem if purified phytate‐degrading enzymes were used. Another significant benefit of postbiotics is their low‐risk profile, as they do not require the ingestion of billions of living bacteria (Shigwedha, 2014). Furthermore, postbiotics may be delivered in a regulated and standardized manner, when utilizing live bacteria, the level of active structure in the gut is determined by the number and metabolic functions of the strain (Pollonio, 2017). So, particular soluble components from certain bacteria may become a type of bacterial biological approach for treating a variety of disorders. While many foodstuffs and their precursors have an abundant amount of postbiotics that are naturally present (e.g., yogurt, kefir, and pickled vegetables) (Chaluvadi et al., 2015). Instead of being manufactured in situ by the producing strain, various postbiotics have been introduced to meals. Cell‐free supernatant from *L. Plantarum YML007* has been researched as a bio preservative on soybean grains (Jung et al., 2013). The huge variety of different food products (dairy or nondairy) present in the market that have bioactive compounds just as probiotics and postbiotics fulfill the dietary requirements of consumers with different nutritional selection, such as some consumers feel allergic to milk proteins and lactose intolerance. It is easy to utilize postbiotics in foods and raw materials before any thermal process without compromising their functions because they are stable throughout different temperatures and pH range. It could benefit technically and economically to the manufacturers (Thorakkattu et al., 2022).

Because of postbiotic's major role in the physicochemical and sensory qualities in the final products, EPS comprising unique sugars have been explored for new applications in the food industry; EPS from lactic acid bacteria, apart from dextran, has not yet been economically utilized as a food additive due to poor outputs (Torino et al., 2015). The only lantibiotic permitted by Food and Drug Administration as a food preservative is nisin, an antibiotic generated by particular *Lactococcus lactis*.

The production of innovative bioengineered probiotic strains that are capable of producing metabolites aimed at the protection and treatment of a variety of disorders has been made possible by the introduction of current genetic manipulation techniques (Sola‐Oladokun et al., 2017). Keeping in view the fact that new antibiotics have been delivered to the intestine using genetically engineered lactic acid bacteria (Amalaradjou & Bhunia, 2013) inhibiting compounds for angiotensin‐converting enzyme (Yang et al., 2015) cancer‐suppressing peptide KiSS1 combining protein of HSP65 with tandem repeats of P277 (Ma et al., 2014), and glutamic acid decarboxylase and IL‐10 cytokine (Huibregtse et al., 2012). These propose potential treatments for resistant bacteria, hypertension, colon cancer, and immune‐related illnesses of the intestine, such as type 1 diabetes. Despite the potential medicinal uses of recombinant probiotic metabolites, important safety and regulatory issues must be solved.

## 8 SAFETY ISSUES OF POSTBIOTICS

Scientific experiments have been conducted to validate the relevant absorption, metabolism, and supply of postbiotics as resolving safety concerns; such compounds can also increase signals to numerous body organs (Tomasik & Tomasik, 2020). Including certain postbiotic safety concerns, a systemic evaluation of seven randomized controlled trials (RCTs) including 1740 infants examined postbiotics' importance in suppressing as well as curing prevalent infectious illnesses in kids under the age of 5 years. In the infants who took inactivated *L. acidophilus* (*LB*) with micronutrient, approximately three of those RCTs evaluated the postbiotic's negative impacts, and some of the documented indirect impacts were a rise in stomach distention, severe dehydration, and vomiting. There were no side effects reported in the other RCTs. Several inquiry studies have highlighted probable negative effects of postbiotic use, according to experts (Malagón‐Rojas et al., 2020). Postbiotic agents possess distinct characteristics like well‐understood chemical compositions, health assessments, longer shelf lives, and resilience in both marketplace and gastrointestinal system settings. They may be safe substitutes for live probiotic cells used in the food and pharmaceutical sectors to provide health benefits, prevent ailments, and achieve therapeutic goals. Physiological characteristics and durability of postbiotic metabolites must be maintained during the preparing stage, and also in delivery methods and hazard aspects.

## 9 CONCLUSION

Postbiotics, even if they are bacterial metabolites or bacterial components, are advantageous and manage to emulate the useful effects of probiotics in therapy. Additionally, they can overcome the systemic administration of viable microbes and treat ailments as such. They have been acknowledged for their immunomodulatory, metabolic, antioxidant, anticancer, and antiobesogenic functions. Postbiotics are researched and experimented with as a revolutionary therapeutic approach both in vitro and in vivo along with the food industry. It is accepted that, in comparison with other biotics, postbiotics have prolonged shelf life besides easier transport, storage, and handling are convenient. Postbiotics are forecasted to pave the way for the development of novel pharmacological and therapeutic or food products with particular and beneficial physiological outcomes. Postbiotic biological responses have been investigated in cell cultures and cell lines, and animal models and confirmations of experimentation were made by the human trials.

## AUTHOR CONTRIBUTIONS

**Abrar Asif:** Writing – original draft (equal); writing – review and editing (equal). **Muhammad Afzaal:** Conceptualization (equal); writing – original draft (equal). **Hina Shahid:** Data curation (equal); writing – review and editing (equal). **Farhan Saeed:** Supervision (equal); writing – review and editing (equal). **Aftab Ahmed:** Formal analysis (equal); validation (equal). **Yasir Abbas Shah:** Writing – original draft (equal); writing – review and editing (equal). **Afaf Ejaz:** Software (equal); validation (equal). **Samia Ghani:** Formal analysis (equal); writing – review and editing (equal). **Huda Ateeq:** Formal analysis (equal); validation (equal). **Mahbubur Rahman Khan:** Formal analysis (equal); validation (equal).

## CONFLICT OF INTEREST STATEMENT

The authors declare no conflict of interest.

## ETHICS STATEMENT

The study involved no experimentation with human subjects.

## CONSENT TO PARTICIPATE

The authors declare their consent to participate in this article.

## CONSENT TO PUBLISH

The authors declare their consent to publish this article.

## Data Availability

Even though adequate data have been given in the form of tables and figures, all authors declare that if more data are required, then the data will be provided on a request basis.
